# Global Biodiversity of Aquatic Ammonia-Oxidizing Archaea is Partitioned by Habitat

**DOI:** 10.3389/fmicb.2012.00252

**Published:** 2012-07-18

**Authors:** Steven J. Biller, Annika C. Mosier, George F. Wells, Christopher A. Francis

**Affiliations:** ^1^Department of Environmental Earth System Science, Stanford UniversityStanford, CA, USA; ^2^Department of Civil and Environmental Engineering, Stanford UniversityStanford, CA, USA

**Keywords:** biogeography, ammonia-oxidizing archaea, nitrification, Thaumarchaeota, *amoA*

## Abstract

Archaea play an important role in nitrification and are, thus, inextricably linked to the global carbon and nitrogen cycles. Since the initial discovery of an ammonia monooxygenase α-subunit (*amoA*) gene associated with an archaeal metagenomic fragment, archaeal *amoA* sequences have been detected in a wide variety of nitrifying environments. Recent sequencing efforts have revealed extensive diversity of archaeal *amoA* sequences within different habitats. In this study, we have examined over 8000 *amoA* sequences from the literature and public databases in an effort to understand the ecological factors influencing the distribution and diversity of ammonia-oxidizing archaea (AOA), with a particular focus on sequences from aquatic habitats. This broad survey provides strong statistical support for the hypothesis that different environments contain distinct clusters of AOA *amoA* sequences, as surprisingly few sequences are found in more than one habitat type. Within aquatic environments, salinity, depth in the water column, and temperature were significantly correlated with the distribution of sequence types. These findings support the existence of multiple distinct aquatic AOA populations in the environment and suggest some possible selective pressures driving the partitioning of AOA *amoA* diversity.

## Introduction

Nitrification – the two-step oxidation of ammonia (NH_3_) to nitrate ${\text{NO}}_{3}^{\text{−}}$ via nitrite ${\text{NO}}_{2}^{\text{−}}$ – is a critical component of the global nitrogen cycle. While bacteria were long thought to be the sole organisms capable of carrying out nitrification, there is now considerable evidence that members of the Archaeal domain are also capable of carrying out the first step of nitrification, the oxidation of NH_3_ to ${\text{NO}}_{2}^{\text{−}}$ (reviewed by Francis et al., 2007). Ammonia-oxidizing archaea (AOA) are now known to comprise a diverse and seemingly ubiquitous group of microorganisms that may make a substantial contribution to the global nitrogen and carbon cycles. Intriguingly, recent studies have shown that the AOA belong to a new phylum of Archaea, the Thaumarchaea (Brochier-Armanet et al., 2008).

The *amoA* gene has proven to be a useful molecular marker for aerobic ammonia oxidizers in the environment. *amoA* encodes the α-subunit of the ammonia monooxygenase enzyme, which catalyzes the initial and rate-limiting step in nitrification. The first suggestion that ammonia oxidation could occur within the Archaea came from the discovery of *amoA* homologs associated with crenarchaeal metagenomic fragments (Venter et al., 2004; Treusch et al., 2005). Definitive evidence for ammonia oxidation capability within the Archaeal domain has come from the successful enrichment and cultivation of AOA, including *Nitrosopumilus maritimus* (Könneke et al., 2005), *Nitrososphaera gargensis* (Hatzenpichler et al., 2008), *Nitrosocaldus yellowstonii* (De La Torre et al., 2008), *Nitrosoarchaeum limnia* (Blainey et al., 2011; Mosier et al., 2012), *Nitrosotalea devanaterra* (Lehtovirta-Morley et al., 2011), *Nitrososphaera viennensis* (Tourna et al., 2011), and *Nitrosoarchaeum koreensis* (Jung et al., 2011).

Numerous studies have provided evidence that archaeal *amoA* sequences are present, and often prevalent, in diverse nitrifying environments such as estuarine sediments, soils, and in the ocean water column (e.g., Francis et al., 2005; Leininger et al., 2006; Park et al., 2006; He et al., 2007; Mincer et al., 2007; Agogué et al., 2008; Beman et al., 2008; Hansel et al., 2008; Mosier and Francis, 2008; Santoro et al., 2008; Jia and Conrad, 2009; Kalanetra et al., 2009; Moin et al., 2009; Wells et al., 2009). Studies have shown that the majority of Marine Group I (MGI) Thaumarchaea contain at least one copy of this gene, underscoring the abundance and likely functional importance of *amoA* in the marine environment (Mincer et al., 2007; Beman et al., 2010; Church et al., 2010; Santoro et al., 2010). While archaeal *amoA* sequences tend to cluster into a few major phylogenetic groups, each of those clades harbors significant fine-scale diversity (Gubry-Rangin et al., 2011; Pester et al., 2012). In environments where the relative diversity of *amoA* sequences from AOA and ammonia-oxidizing bacteria (AOB) have been compared, AOA *amoA* diversity is usually observed to be much higher than that of AOB *amoA* (e.g., Mosier and Francis, 2008; Santoro et al., 2008; Wankel et al., 2011), although the reasons for this difference are not clear.

Most AOA are thought to be chemolithoautotrophs, but recent work in wastewater treatment plants has suggested that some may be capable of oxidizing organic compounds as energy and carbon sources (Mussmann et al., 2011). Field studies have demonstrated the functional importance of Thaumarchaea in ammonia oxidation in both aquatic and soil environments (Leininger et al., 2006; Lam et al., 2009; Santoro et al., 2010; Zhang et al., 2010), although the presence of archaeal *amoA* genes has not always been found to correlate with active nitrification *in situ* (Mussmann et al., 2011). The relationship between the relative abundance and activity of AOA and AOB appears to be complex, and depends at least in part on salinity and other environmental variables (e.g., Erguder et al., 2009, and references therein).

Inspection of *amoA* phylogenies has suggested that sequences tend to cluster with others from the same environment, but the significance of this association has remained largely unclear. In this study, we sought to determine the importance of habitat type in explaining *amoA* phylogeny and to investigate environmental and ecological factors that may be responsible for partitioning AOA *amoA* genotypic diversity. To this end, we used a bioinformatic approach to characterize the diversity and distribution of over 8000 AOA *amoA* sequences from the GenBank database, with a particular focus on *amoA* sequences from aquatic habitats. Multiple independent approaches, including a complete phylogenetic analysis of the entire dataset of unique sequences, support the hypothesis that different environmental habitats harbor distinct and largely coherent groups of *amoA* sequences. We find a significant role for salinity, temperature, and other environmental parameters in partitioning AOA diversity in aquatic habitats. Together, these results indicate the existence of largely distinct populations of AOA occupying different habitats in the environment.

## Materials and Methods

### *amoA* sequence dataset

Archaeal *amoA* sequences were extracted from GenBank (release 175) by searching for records identified as environmental samples containing the search terms “*amoA*” and “uncultured ammonia-oxidizing archaeon,” “uncultured archaeon,” or “uncultured crenarchaeote.” Metadata for each sequence was either downloaded directly from GenBank or manually annotated by referring to the original publication (where available). We assigned each sequence to one of 13 habitat categories: aquaria and biofilters, caves, coastal sediments, coral and sponges, groundwater (including groundwater treatment), hot springs, hydrothermal vents, lakes and rivers, marine sediments, seas, soils, water column (marine), and wastewater treatment (including wastewater treatment plants, activated sludge, and bioreactors).

Archaeal *amoA* sequences were aligned in ARB (Ludwig et al., 2004) using a seed-alignment constructed in MEGA v4.0.2 (Tamura et al., 2007) based on nucleotide sequences. Four partial-length betaproteobacterial ammonia-oxidizing bacterial *amoA* sequences [*Nitrosomonas europaea* (AF058691), *Nitrosospira briensis* (U76553), *Nitrosospira multiformis* (AF042171), *Nitrosomonas cryotolerans* (AF314753)], one partial-length gammaproteobacterial ammonia-oxidizing bacterial *amoA* sequence [*Nitrosococcus oceanus* (AF047705)], and two partial-length methane-oxidizing bacterial *pmoA* sequences [*Methylosinus trichosporium* OB3b (U31650) and *Methylococcus capsulatus* (L40804)] were aligned against amino acid translations of selected archaeal *amoA* sequences in Geneious v4.8.5 (Biomatters Ltd., Auckland, New Zealand) for use as an outgroup. The alignment was trimmed to a final length of 534 bp to maximize the number of sequences included in the final dataset while still removing low-quality sequence ends. Sequences of insufficient length (e.g., DGGE bands) or with insufficient metadata were discarded.

### Phylogenetic tree construction

Phylogeny was inferred with RAxML v7.2.6 (Stamatakis, 2006) as implemented in the CIPRES portal (Miller et al., 2009). Two-hundred independent maximum likelihood inferences were run on the alignment, starting from independent randomized maximum parsimony trees; the best-scoring maximum likelihood tree was used as the final tree. Habitat associations (based on 13 manually defined categories) were mapped onto the tree using the interactive Tree of Life (iTOL) program (Letunic and Bork, 2007).

### Bioinformatic analyses

MOTHUR v1.11.0 (Schloss et al., 2009) was used to determine the number of operational taxonomic units (OTUs) present in the AOA *amoA* dataset at varying levels of sequence identity, calculate rarefaction curves, and compute beta-diversity metrics between sequences from different habitats. LIBSHUFF analysis was also performed from within MOTHUR, using the default settings. Calculations of the Jaccard index for protein sequences were performed using a custom Python script. Due to the nature of this dataset, we did not have sufficient abundance information to calculate quantitative indices of alpha or beta-diversity that incorporate species richness information.

Average pairwise identities between sequences at both the nucleotide and amino acid level were carried out using a custom Python script. Tests for selection pressures on AmoA were conducted using the maximum likelihood-based SLAC methodology (Kosakovsky Pond and Frost, 2005) as implemented in the HyPhy package (Pond et al., 2005) and run using the web interface at http://www.datamonkey.org (Pond and Frost, 2005). To look for evidence of selection in the overall dataset, we analyzed representative sequences of OTUs at the 85% identity level as determined by MOTHUR; this was done both due to computational limitations and to increase the likelihood that the sequences being analyzed represented fixed lineages from distinct populations, and not simply polymorphisms within a population (Kryazhimskiy and Plotkin, 2008). For analyses within a habitat type, sequences representing the 90% identity OTUs were used to ensure that a sufficient number of sequences were analyzed. Automatic nucleotide substitution model selection and recombination detection (using GARD, when possible) were both carried out before the SLAC analysis.

Sequences assigned to the “coastal sediments” and “lakes and rivers” habitats were analyzed further using AdaptML (Hunt et al., 2008). AdaptML defines ecologically meaningful phylogenetic groups using an evolutionary hidden Markov model that identifies populations as groups of related strains sharing a common projected habitat. The default parameters were used except for our use of a more precise numerical optimization for the habitat transition rate parameter. Sequences were assigned to a habitat sub-category based on the metadata: coastal, surf zone, estuary, salt marsh, lake, heathland pool, or river. Additionally, each sequence was assigned to a high (≥15 ppt) or low (<15 ppt) salinity category. Clonal sequences were removed from the sequence dataset, except where unique sequences were found in more than one habitat type. Out of 2470 total sequences in the broad coastal sediment, lake, and river habitat categories, 1997 sequences were used in the AdaptML analysis (1962 unique sequences and 35 additional non-unique sequences representing a different sub-category). Phylogenetic trees used as an input into AdaptML were reconstructed using PhyML v.2.4.4 using the ATGC bioinformatics platform (Guindon and Gascuel, 2003) with the following parameter settings: DNA substitution was modeled using the HKY parameter; the transition/transversion ratio was set to 4.0; PhyML estimated the proportion of invariable nucleotide sites; the gamma distribution parameter was set to 1.0; 4 gamma rate categories were used; a BIONJ tree was initially used; and branch lengths and rate parameters were optimized by PhyML. *Nitrosomonas europaea* (AF058691) was used as an outgroup. AdaptML output files were visualized using iTOL (Letunic and Bork, 2007).

### Statistical analyses

Principal components analysis was carried out using the unweighted FastUnifrac algorithm on the Unifrac website (http://bmf2.colorado.edu/fastunifrac/index.psp; Hamady et al., 2010), using the best RAxML tree and a file assigning each sequence to one of 13 different habitat groupings as input. Analyses were carried out using the default parameters or as indicated in the text. ANOSIM and perMANOVA analyses were conducted with 1000 permutations in the R statistical programming environment v2.11.1 using algorithms implemented in the package vegan v1.17 (Oksanen et al., 2010) with distance matrices generated in MOTHUR v1.11.0 and associated metadata as inputs. Experimental factors tested were derived from sequence metadata and included habitat (categories as described above), temperature (psychrophilic, mesophilic, and thermophilic, defined as <15°C, between 15 and 40°C, and >40°C, respectively), salinity (low and high, defined as <15 and ≥15 ppt, respectively), latitude (low-, mid-, and high-latitude, defined as <23.4°, between 23.4° and 66.6°, and >66.6°, respectively), and ocean water column depth (surface, mid, and deep, corresponding to <199 m, between 200–399 m, and ≥400 m depth, respectively).

## Results and Discussion

### Overview of archaeal *amoA* sequence diversity

**Figure A1:**
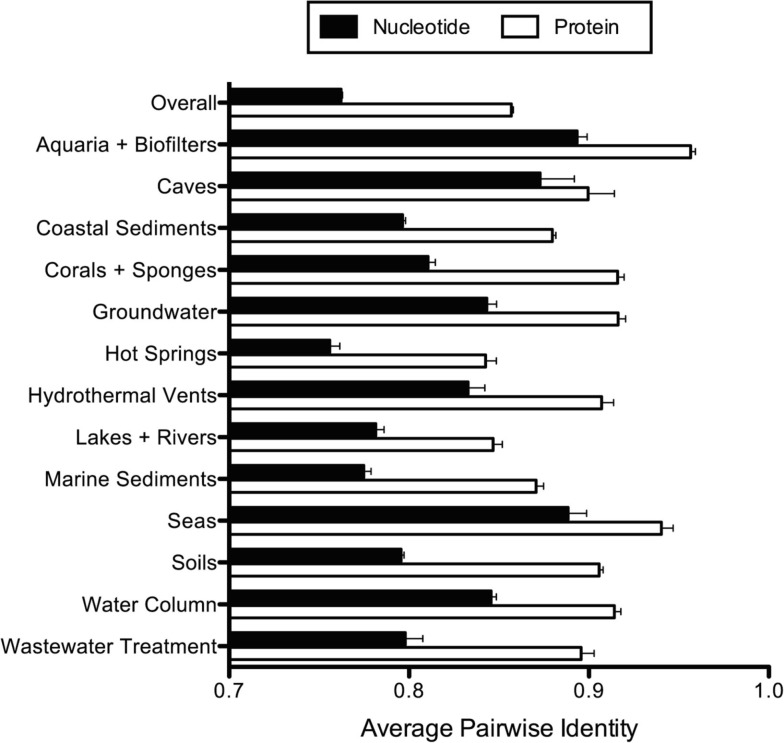
**Average pairwise *amoA* nucleotide and protein sequence identity**. Error bars represent the standard error of the mean.

We compiled and aligned 8296 archaeal *amoA* sequences from GenBank, representing nearly 100 different environmental- and cultivation-based studies (published and unpublished) from around the world. The vast majority of *amoA* sequences came from coastal sediments (~30%) and soils (~32%). Caves, seas, hydrothermal vents, and wastewater treatment were the most underrepresented habitats in terms of total number of sequences in the database (Table 1). Of the 8296 total amoA sequences within our alignment, 6203 (~75%) were unique. On average, *amoA* sequences were 76% identical to each other at the nucleotide level (Figure A1 in Appendix; Table 1). Although some of this apparent “diversity” could arise from experimental artifacts, we note that even allowing for ~5 PCR- or sequencing-based errors in each *amoA* fragment (corresponding to the 99% identity level) still yields extensive diversity among AOA (Table 1).

**Table 1:** **Summary statistics for the AOA *amoA* sequence dataset**.

	Total sequences	Number of nucleotide sequence OTUs (identity level)					Unique protein sequences
		Unique	99%	95%	90%	85%	
Overall	8296	6203	2494	805	315	138	3729
Aquaria + Biofilters	277	159	48	18	8	4	104
Caves	82	52	6	5	4	3	41
Coastal sediments	2459	1947	864	355	150	66	1296
Coral + Sponges	407	282	101	56	42	30	194
Groundwater	215	180	81	26	15	10	123
Hot springs	197	183	121	80	51	31	169
Hydrothermal vents	120	95	43	25	17	12	77
Lakes + Rivers	445	355	150	72	39	27	228
Marine sediments	342	299	232	125	65	41	243
Seas	95	73	22	8	6	5	52
Soils	2621	1748	704	280	137	70	1031
Water column	902	767	301	64	27	16	304
Wastewater treatment	134	100	36	25	20	17	84

**Figure A2:**
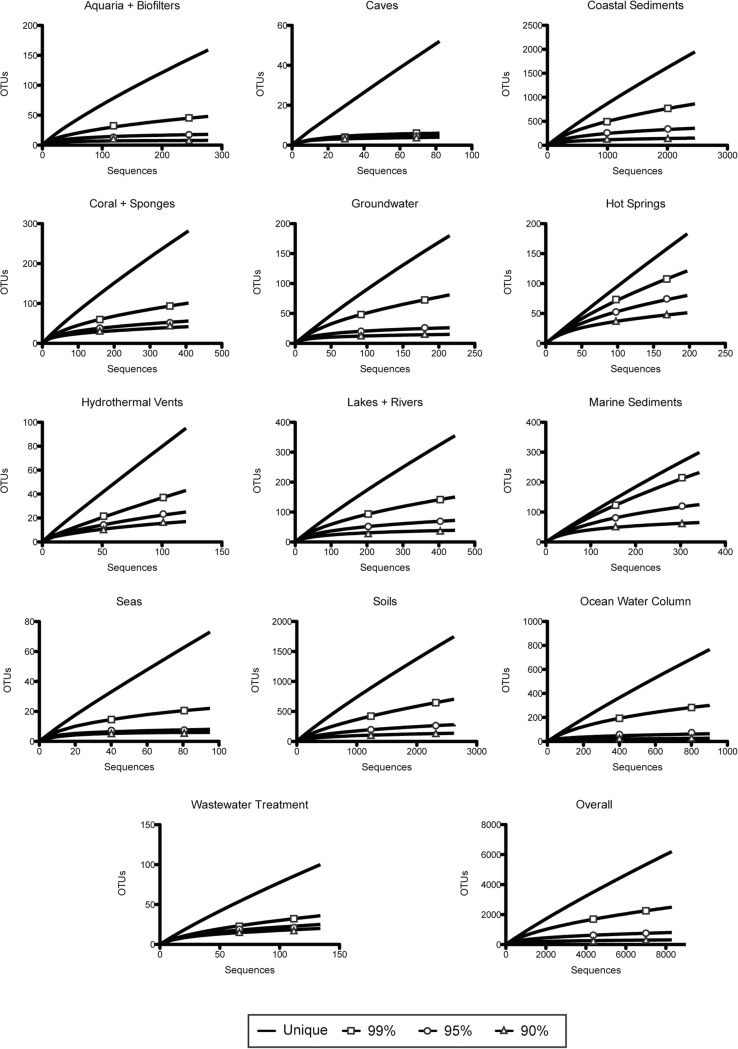
**Rarefaction analysis of *amoA* sequence diversity, by habitat type**. Curves represent rarefaction analysis of unique sequences and OTUs defined by 99, 95, and 90% identity.

Pester et al. (2012) proposed that *amoA* sequences with less than 87% nucleic acid sequence identity are likely to represent two different AOA species. Bracketing this value, when grouping sequences at a 90% nucleotide identity level, we identified 315 AOA “species” across all environments sampled; at 85% identity, we observed 138 *amoA*-based “species” (Table 1). Although the degree of genetic diversification reflecting species differentiation is controversial (for both 16S rRNA genes and functional genes such as *amoA*), these numbers reflect a general estimate of the overall diversity of AOA across wide-ranging environments. Although the observed number of OTUs decreased rapidly from the 99% identity level to the 95 and 90% identity levels, rarefaction analysis indicates that the unique diversity of *amoA* sequences observed thus far is far from saturating (Figure A2 in Appendix). Sequences from coastal sediments have the highest number of observed OTUs (at all identity levels), followed by soils. Caves and seas have the fewest observed OTUs.

The diversity of *amoA* sequences from hot springs and marine sediments appear to be the most undersampled, based on the high percentage of unique nucleotide sequences and OTUs (at all levels) relative to the total number of sequences (Table 1). Conversely, rarefaction analysis suggests that the diversity within aquaria and caves appear to be the most oversampled (Figure A2 in Appendix); however, it is important to note that only a few studies have looked at AOA from these environments. It is possible, if not likely, that additional data from other types of aquaria, biofilters, or caves would increase the diversity of sequences observed within these groups. While the primers used to amplify *amoA* can potentially affect the relative diversity observed in these habitats, our analysis did not exclude any particular primer set. Overall, AOA *amoA* diversity appears to have been well sampled in the literature, but this analysis suggests that the number of undiscovered *amoA* sequence types in the environment is still potentially vast.

### Different environments contain distinct groups of *amoA* sequence types

**Figure 1:**
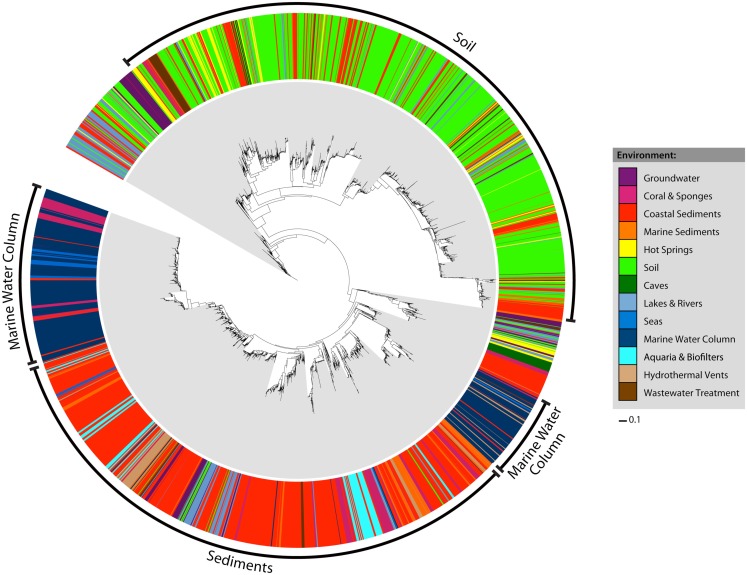
**Maximum likelihood phylogeny and habitat distribution among all 6203 unique AOA *amoA* sequences**. Colored bars in the outer ring correspond to the habitat assignment for each individual sequence. The bacterial *amoA* out group has been excluded from the tree for visualization purposes.

Early studies of archaeal *amoA* diversity (Francis et al., 2005) suggested that *amoA* sequences from sediment and water column samples formed distinct phylogenetic clusters. We sought to determine whether this pattern could still be observed in a more current and significantly larger *amoA* dataset. Habitat assignments were mapped onto a maximum likelihood phylogenetic tree of all 6203 unique *amoA* sequences (Figure 1). Four major clusters were evident in the tree: one representing sequences from coastal and marine sediments, one from soils, and two groups from the marine water column. While the groups were not explicitly defined by habitat definition, the sequences were strongly correlated with environment. For example, within the two marine water column clusters, nearly all sequences that did not come from that environment instead came from other marine habitats: corals, seas, hydrothermal vents, and marine sediments. Water column sequences were rarely observed outside of the two water column groups. Coral sequences fell predominantly in the sediment and water column cluster. Most of the hot spring and wastewater treatment sequences grouped within the soil cluster, whereas most of the other habitat types grouped predominantly within the sediment cluster. Statistical analyses support the idea that there is a strong correlation between *amoA* phylogenetic and ecological differentiation. Both a *P*-test and overall Unifrac significance test indicated that there was significant clustering of sequences as grouped by habitat on the tree (*P*-test *P* = 0; Unifrac test *P* < 0.001). In addition, each of the 13 habitat-defined groups of *amoA* sequences was significantly different from all others by the LIBSHUFF test (*P* < 0.0001).

We used Unifrac distances between *amoA* sequences from each habitat to better understand the phylogenetic relationships between these groups. Unifrac provides a measure of the amount of evolution in a tree that is confined to a specific habitat grouping, or how much more unique branch length is attributed to a particular environment than would be expected by chance. Analysis of Unifrac distances between individual habitat types indicated that sequences found in coastal sediments, corals and sponges, groundwater, hot springs, marine sediments, and soils were significantly clustered on the phylogenetic tree (*P* < 0.001 for all except groundwater, *P* = 0.013). This result corroborates previous findings that suggested the presence of distinct groupings from coastal sediments and soil habitats (e.g., Francis et al., 2005) and supports the hypothesis that different environmental habitats tend to contain distinct groups of *amoA* sequence types. In contrast to the LIBSHUFF results, *amoA* sequences from aquaria and biofilters, caves, hydrothermal vents, lakes, and rivers, seas, wastewater treatment, and the water column categories were not distinct from all others by the Unifrac test. This may reflect artifacts of our metadata-based habitat definitions not truly reflecting environmentally relevant groupings, the number of sequences examined per category, or result from some shared selective pressures on *amoA* among these groups.

β-diversity analysis of the distribution of *amoA* OTUs also indicated that *amoA* sequence types are strongly partitioned by environment. Pairwise comparisons of the number of OTUs shared between any two habitats (using the Jaccard index) showed that, even at a coarse 90% identity level, many environments had no OTUs in common; at most, 23% of the total OTUs observed in any two habitats were found in both (Table 2). The proportion of shared OTUs dropped markedly at the 95 and 99% identity level (less than 14 and 3%, respectively, of the total OTUs found in any two habitats were shared; Table A1 in Appendix). ANOSIM (*R* = 0.378, *P* = 0.001) and perMANOVA (*R*^2^ = 0.221, *P* = 0.001) analyses confirmed highly significant habitat partitioning among archaeal *amoA* sequence types (Table 3; Table A2 in Appendix). Taken together, these results suggest that groups of AOA (as defined by their *amoA* sequence) found in different environments are significantly different from one another.

**Table 2:** **β-Diversity among environment types**.

	Aquaria + biofilters	Caves	Coastal sediments	Coral + sponges	Groundwater	Hot springs	Hydrothermal vents	Lakes + rivers	Marine sediments	Seas	Soils	Ocean water column	Wastewater treatment
Aquaria + biofilters		0	0.053	0.087	0.045	0	0.042	0	0.090	0.077	0	0.094	0.037
Caves	0		0.007	0	0	0.058	0	0	0.015	0	0.007	0	0
Coastal sediments	0.008	0.001		0.129	0.058	0.117	0.037	0.167	0.229	0.033	0.221	0.099	0.097
Coral + sponges	0	0	0.007		0	0	0.054	0	0.081	0.067	0	0.113	0.016
Groundwater	0	0	0.006	0		0.031	0	0.125	0.039	0	0.048	0	0.029
Hot springs	0	0.014	0.005	0	0		0	0.233	0.084	0	0.182	0.013	0.109
Hydrothermal vents	0	0	0	0.007	0	0		0	0.206	0.095	0	0.158	0
Lakes + rivers	0	0	0.014	0	0.014	0.018	0		0.118	0	0.205	0.015	0.157
Marine sediments	0.009	0	0.018	0.002	0.003	0.01	0.022	0.015		0.044	0.092	0.136	0.076
Seas	0	0	0.001	0.008	0	0	0.008	0	0.003		0	0.179	0
Soils	0	0	0.022	0.001	0.004	0.008	0	0.021	0.014	0		0.006	0.090
Ocean water column	0.005	0	0.006	0.020	0	0.002	0.016	0.002	0.009	0.017	0.001		0.044
Wastewater treatment	0	0	0.005	0	0	0.008	0	0.010	0.009	0	0.005	0.003	

*Values represent the Jaccard similarity index for (above the diagonal) *amoA* nucleotide OTUs at the 90% identity level and (below the diagonal) unique AmoA protein sequence types*.

**Table 3:** **Environmental determinants of archaeal *amoA* sequence diversity**.

Factor	Sequences included in analysis		
	All sequences with associated metadata (*n* = 2014)^1^	Marine water column sequences (*n* = 290)^2^	Aquatic sequences (water column, groundwater, sea, lakes/rivers; *n* = 984)^3^
Habitat (13 levels)	**0.221**	NA	NA
Latitude (low, mid, high)	**0.010**	**0.092**	**0.057**
Temperature (pychrophilic, mesophilic, thermophilic)	**0.017**	**0.023**	NA
Water depth (surface, mid, deep)	NA	**0.335**	NA
Salinity (high, low)	NA	NA	**0.197**
Habitat and temperature	**0.017**	NA	NA
Habitat and latitude	**0.012**	NA	NA
Temperature and latitude	0.0005	0.003	
Depth and latitude	NA	**0.019**	NA
Temperature and depth	NA	**0.016**	NA
Latitude and salinity	NA	NA	**0.023**
Total variation explained by combined factors	0.248	0.469	0.277
Residual variation	0.723	0.531	0.723

**R*^2^ values for factors tested via perMANOVA for association with variation in *amoA* diversity are indicated; significant *R*^2^ values (*P* < 0.001) are highlighted in bold. Analyses were carried out on the indicated subset of sequences for which the relevant metadata was available. Level designations for each habitat are provided; definitions for levels are given in the text. For each sequence grouping, all factors were concurrently subjected to perMANOVA. *R*^2^ indicates the proportion of variation each factor contributes to the total variation in the dataset*.

*NA, not applicable*.

*^1^Of 6203 unique archaeal *amoA* sequences, both latitude and temperature data were available for 2014*.

*^2^Of 902 sequences classified as water column, latitude, depth, and temperature metadata were available for 290*.

*^3^Of 1657 water-associated sequences, latitude and salinity metadata were available for 984. Temperature designation was not included in this perMANOVA due to the fact that all 290 available sequences with temperature sub-category assignments were classified as water column sequences, and thus the perMANOVA analysis with temperature would be identical to the Water Column-specific analysis (the middle column in the table)*.

### Potential for *amoA* functional diversity in different environments

The existence of distinct *amoA* sequences in different environments could be explained by varying selective pressures on the function of the AMO enzyme complex. To explore the potential for functional diversity within the ammonia monooxygenase α-subunit, we examined the distribution of AmoA sequence types at the amino acid level. The 6203 unique *amoA* sequences in our dataset yielded 3729 unique protein variants, which had an average pairwise identity of 86% (Figure A1 in Appendix). Interestingly, no amino acid position was completely conserved across the entire *amoA* dataset. When comparing sequences from individual habitats, we found that AmoA was most similar (96%) within each of the aquaria/biofilters and sea categories; this may be attributable to the relatively constant nature of these environments. The lowest average pairwise identities within an environment were 84 and 85% from sequences in hot springs and lakes/rivers, respectively. We note that there was no correlation between the number of unique protein sequences sampled in an environment and the average amino acid identity (*R*^2^ = 0.03). Qualitative ß-diversity analysis of the translated AmoA protein sequences indicated that, like the nucleotide sequences, biodiversity at the amino acid level was strongly partitioned within each habitat type; no more than ~2% of AmoA protein sequences were shared between two or more habitats (Table 2).

To gain insight into the strength of selective pressures acting on AmoA, we calculated the ratio of non-synonymous to synonymous substitutions (dN/dS) in our dataset. Using sequences representing the 90% OTUs, the dN/dS ratio for *amoA* was 0.048; this indicates that deleterious *amoA* mutations are removed from archaeal populations through purifying (negative) selection. Furthermore, there was no evidence for positive selection at any individual codon position in our alignment (SLAC algorithm; *P* < 0.05).

We were curious whether the selective pressures acting upon ammonia-oxidation functions might differ among environments. To address this, we calculated dN/dS ratios for AmoA sequences from each of the broad habitat categories (Figure A2 in Appendix). While the dN/dS value was indistinguishable from the overall value in many environments, there was a small but significant increase in dN/dS (i.e., weaker purifying selection) on *amoA* sequences from five environments: caves, groundwater, hot springs, hydrothermal vents, and the marine water column (95% confidence intervals do not overlap with the value from the overall dataset). The relaxation of purifying selection (or increased positive selection) in these five environments could arise from changes in environmental conditions selecting for functional differences in AmoA, or a change in the expression levels or selective importance of AmoA to the overall fitness of archaea in these environments. These differences could also reflect changes in the effective population sizes that might affect the efficiency of purifying selection in these environments.

### Environmental parameters influence the distribution of *amoA* sequence types

**Figure 2:**
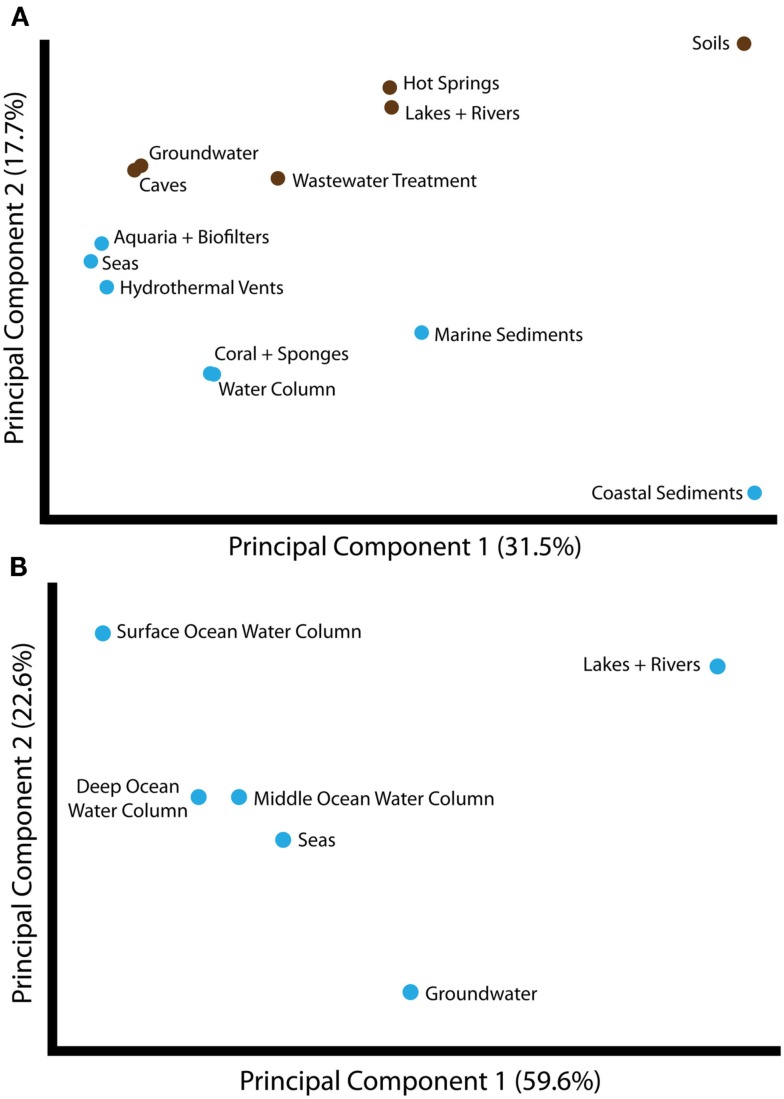
**Principal components analysis of unweighted Unifrac distances between different groups of *amoA* sequences, as categorized by habitat from which the sequences were isolated**. **(A)** Unifrac analysis of *amoA* sequences from all habitats annotated in the complete *amoA* sequence dataset. The first two principal components account for 49% of the variation between all habitats. Unifrac analysis was conducted based on the maximum likelihood tree shown in Figure 1. Habitats are colored as either marine (blue) or freshwater/terrestrial (brown). **(B)** Unifrac analysis of *amoA* sequences from aquatic environments only. A maximum likelihood tree was computed for all unique sequences from the indicated habitats using RAxML and analyzed by Unifrac as above. The major principal component correlates with salinity.

Given the evidence for partitioning of *amoA* sequence diversity by habitat type, we wanted to understand the environmental factors that might be principally responsible for driving these patterns. As a first step, we compared the groupings of *amoA* sequences from different habitats using Unifrac. The largest component of the unweighted Unifrac distances between environments generally separated samples from soil- or sediment-associated environments versus samples from aquatic habitats (Figure 2A). Principal component 2 corresponded to a division between sequences from marine and freshwater/terrestrial environments. ANOSIM analyses confirmed a strong and significant divergence between sequences associated with marine versus freshwater/terrestrial environments (*R* = 0.422, *P* < 0.001) and moderate divergence between sequence groupings associated with aquatic versus soil or sediment environments (*R* = 0.099, *P* < 0.001; Table A2 in Appendix).

Surveys of AOA *amoA* diversity in soils have shown that different groups of *amoA* sequences are found in different geographic locations (Gubry-Rangin et al., 2011; Pester et al., 2012). Although many sequences lacked detailed location data, we asked whether two broad categories generally associated with geographic variation – temperature and latitude – were correlated with sequence diversity in our dataset. ANOSIM analyses indicated a moderate association with temperature, and a weak but significant association with latitude (Table A2 in Appendix). The perMANOVA analyses found that the combination of habitat type, latitude, and temperature could explain approximately 25% (*R*^2^ = 0.248) of the variance in our global alignment, but the vast majority of this variance was accounted for by habitat type (Table 3). While latitude and temperature were both significantly associated with variation in sequence type (*P* < 0.001), they could account for a mere 1.7 and 1% of variance in sequence diversity, after accounting for the influence of habitat type. Interaction effects for these factors could account for an additional 2.7% of sequence variation. When assessed independently from habitat type, temperature, and latitude explain 9.7 and 3.4%, respectively, of *amoA* sequence variation. In total, these results suggest that the observed diversity among AOA *amoA* across all environments is linked in part to biogeographic (latitudinal) variation (corroborating the findings of Pester et al., 2012) and temperature, but these factors likely play relatively minor roles compared to the influence of habitat type. Our analysis was limited to metadata available in GenBank or the associated publications and, therefore, it is certainly possible that other environmental variables not analyzed here are correlated with sequence diversity in the overall dataset.

We wanted to determine what environmental parameters might specifically explain the differences in AOA from distinct aquatic habitats, where the associated environmental metadata was most complete. To address this question, we examined the relative importance of depth, salinity, and other environmental factors that could potentially drive *amoA* sequence divergence. We began by examining the phylogenetic relationship between *amoA* sequences from the marine water column, seas, lakes and rivers, and groundwater samples. A global Unifrac significance test on the phylogenetic relationship among these aquatic *amoA* sequences confirmed the earlier inference that there is significant clustering of these environmental groupings (*P* < 0.001).

To assess the potential for differences between *amoA* sequences from different depths in the ocean, we further separated water column sequences into three subgroups: surface (0–199 m), middle (200–399 m), and deep (≥400 m). We found that depth can explain ~30% of the sequence variation in the marine water column (perMANOVA *R*^2^ = 0.335, *P* < 0.001; Table 3). The correlation between *amoA* groupings and ocean depth might be explained by multiple selective factors such as ammonium availability, competition with other organisms, light levels, oxygen concentrations, and physical partitioning of these populations that may have reduced the opportunity for migration. Sequences from surface waters were separated from sequences found at middle or deep depths by the second principal component of variation in the Unifrac distance matrix (Figure 2B); the third principal component roughly correlated with depth among the water column sequences, and explained 11% of the variation within *amoA* sequences from all aquatic habitats. This result is in keeping with previous studies arguing that archaeal *amoA* sequences from the open ocean fall into two phylogenetically distinct groups corresponding to surface and deep water ecotypes (Francis et al., 2005; Hallam et al., 2006; Mincer et al., 2007; Beman et al., 2008; Santoro et al., 2010; Hu et al., 2011; Mosier and Francis, 2011). Given the current level of known *amoA* sequence diversity in the oceans, it is apparent that two major water column ecotypes exist; however, other still-unknown factors in addition to depth are influencing the distribution of these sequence types. Indeed, perMANOVA tests also indicated that the combination of latitude and temperature could account for 37% of sequence variation amongst water column sequences (*P* < 0.001).

Principal components analysis of the Unifrac distances between each group of aquatic *amoA* sequences revealed that 60% of the variation could be explained by an axis that roughly corresponded to salinity (Figure 2B). *amoA* Sequences from freshwater environments (lakes and rivers) formed a phylogenetic cluster that was significantly different from all other groups of aquatic sequences from saline environments (*P* < 0.002; pairwise Unifrac significance test). ANOSIM (*R* = 0.576, *P* < 0.001; Table A2 in Appendix) and perMANOVA (19.7%; Table 3) confirmed that salinity was strongly associated with *amoA* sequence clustering amongst the aquatic habitats. However, a large percentage of the total variation in the aquatic *amoA* dataset could not be explained by salinity alone, suggesting that additional environmental variables significantly influence sequence variation in aquatic environments.

Salinity is a major determinant of overall microbial community composition in the environment (Lozupone and Knight, 2007), and also influences the abundance of AOA in some estuary and surf zone sediments (Mosier and Francis, 2008; Santoro et al., 2008). To further explore whether phylogenetic clustering of AOA *amoA* was associated with salinity, we employed AdaptML (Hunt et al., 2008) to define ecologically coherent populations (ecotypes) amongst the coastal sediment, lake, and river sequences. AdaptML is an evolutionary model that defines ecotypes or inferred habitats within the dataset based on the environmental characteristics of the sampling location (e.g., salinity and environmental setting) and the observed phylogeny. Each sequence was assigned to a high (≥15 ppt) or low (<15 ppt) salinity category based on the reported data from the sampling location. Additionally, the coastal sediment, lake, and river sequences were assigned to sub-categories based on environmental setting: coastal, surf zone, estuary, salt marsh, lake, heathland pool, or river.

**Figure 3:**
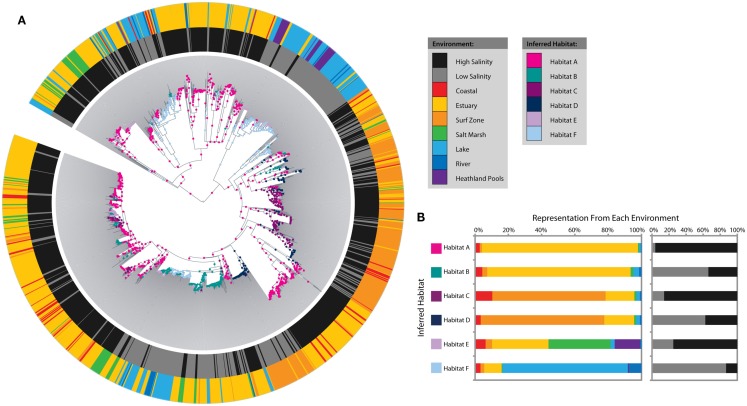
**Salinity and microenvironment ecotype predictions amongst coastal sediment, lake, and river AOA *amoA* sequences as inferred by AdaptML**. **(A)** Maximum likelihood phylogenetic tree showing environmental characteristics and habitat predictions. Characteristics of the sampling locations are plotted on the inner and outer rings: the inner ring indicates high (≥15 ppt) or low (<15 ppt) salinity and the outer ring indicates the microenvironment. Ecological habitats predicted by the model are shown as nodes on the tree. **(B)** The distribution of each habitat among microenvironments and salinity. The habitat and environment colors match the legend in **(A)**.

The analysis identified six distinct ecotypes or inferred habitats with strong signals from salinity and environmental setting (Figures 3A,B). Habitat A was dominated by estuary sequences from high-salinity sites, whereas Habitat B mainly represented estuary sequences from low-salinity sites. Habitats C and D showed a similar salinity distinction amongst surf zone sequences. Habitat E was more cosmopolitan in nature and combined high-salinity sequences from estuaries, salt marshes, and heathland pools. Habitat F corresponded to low-salinity lake sequences. Habitat subcategories (coastal, surf zone, estuary, salt marsh, lake, heathland pool, or river) alone could explain more than 10% of the variation in *amoA* sequence diversity, based on perMANOVA analysis (*R*^2^ = 0.103, *P* < 0.001), while salinity and interaction effects with habitat subcategories explained an additional 2 and 1.1%, respectively.

### Considerations and future areas of research

Previous work has noted the existence of distinct groups of *amoA* sequences associated with different environments, such as soil- and water-associated phylogenetic clades. In this study, we inquired whether the phylogenetic relationships between *amoA* groups and ecological habitats were simply an artifact of early limited sampling or instead reflected a broad trend separating AOA communities in the environment. Our analysis of an aggregated global *amoA* sequence dataset highlights the immense diversity of this gene in the environment and demonstrates that distinct groupings of phylogenetically related *amoA* sequences are indeed found in particular habitats; however, with increased sampling of *amoA* diversity, nuances have begun to emerge.

We find a strong distinction between sediment/soil-associated *amoA* sequence types and those found in other aquatic systems. Factors affecting *amoA* diversity within soils, such as pH, have been discussed extensively elsewhere (Gubry-Rangin et al., 2011; Pester et al., 2012) and were not the focus of this study. Among *amoA* sequences from aquatic ecosystems, environmental factors such as salinity, temperature, and depth in the water column appear to play a role in selecting for and/or maintaining *amoA* sequence diversity. We do not yet know whether the environmental variables studied here are selecting specifically for variants of AmoA with specific functional differences, or are instead selecting on corresponding differences in the rest of the AMO enzyme or associated pathways. It is also possible that *amoA* could play a role in other cellular functions besides ammonia oxidation, and that this could in turn affect the selective pressures on this gene. We also note that the effects of recent recombination and horizontal gene transfer could complicate interpretation of the ecological clustering.

We found that one of the major barriers in trying to place sequence diversity into an environmental context was the lack of standardized environmental metadata from the sites sampled (e.g., salinity, temperature, latitude and longitude, nutrient concentrations, dissolved oxygen concentration, pH, among others). We support efforts to establish a set of standardized metadata submission requirements to accompany sequences from environmental marker genes such as MIMARKS (Yilmaz et al., 2011). A better understanding of the exact selective factors that influence the distribution of *amoA* sequences in different habitats will require such annotations; even still, environmental factors beyond the scope of standardized metadata (e.g., competition for ammonia with other organisms, abiotic processes, etc.), including the range of physicochemical parameters experienced at a given sampling location, likely play a role in shaping the overall distribution and activity of AOA. These limitations argue strongly for the necessity of additional studies on cultivated AOA (both pure isolates and enrichment cultures), to better understand the functional differences between different *amoA* sequence types and the specific factors that drive AOA niche differentiation.

While there is clearly a large amount of diversity at the AmoA protein level, the functional implications of these protein variants is impossible to know at this time. The successful culturing of AOA from different environments provides hope that, in time, physiological studies could shed some light on the functional differences between *amoA* alleles, interactions between mutations within different subunits of the AMO complex, the fitness advantage conferred by different alleles, and the effect of other factors (e.g., gene expression patterns) on the selection of different *amoA* sequences in different habitats.

The dramatic advances in sequencing technology in recent years will undoubtedly allow future studies to more thoroughly survey the diversity of *amoA* sequences in the environment. Our results suggest that the novel *amoA* sequences are most likely to be found in hot springs and marine sediments. Future use of single-cell genomics approaches to uncover other genomic features that covary with *amoA* sequence types will likely lead to a better understanding of the functional context and implications of this diversity, as well as the relative roles of selection, mutation, migration, and other processes in partitioning *amoA* diversity.

## Conflict of Interest Statement

The authors declare that the research was conducted in the absence of any commercial or financial relationships that could be construed as a potential conflict of interest.

## Supplementary Material

The Supplementary Material for this article can be found online at http://www.frontiersin.org/Aquatic_Microbiology/10.3389/fmicb.2012.00252/abstract

## Figures and Tables

**Figure A3:**
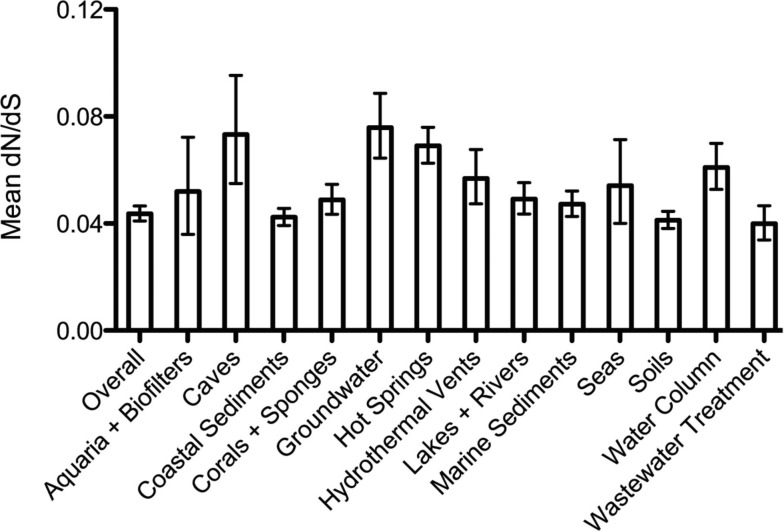
**Selective pressure (dN/dS) on *amoA* sequences from different habitats as calculated by the SLAC algorithm**. Error bars represent 95% confidence intervals.

## Appendix

**Table A1:** **β-Diversity between environment types**.

	Aquaria + biofilters	Caves	Coastal sediments	Coral + sponges	Groundwater	Hot springs	Hydrothermal vents	Lakes + rivers	Marine sediments	Seas	Soils	Ocean water column	Wastewater treatment
Aquaria + biofilters		0	0.010	0	0	0	0	0	0	0	0	0	0
Caves	0		0	0	0	0	0	0	0	0	0	0	0
Coastal sediments	0.042	0		0.009	0.004	0.004	0	0.013	0.013	0.003	0.031	0.010	0.008
Coral + sponges	0.028	0	0.046		0	0	0	0	0	0.008	0	0.023	0
Groundwater	0	0	0.024	0		0	0	0.022	0	0	0.004	0	0.009
Hot springs	0	0.037	0.041	0	0.029		0	0.019	0.006	0	0.015	0	0.006
Hydrothermal vents	0	0	0.008	0.013	0	0		0	0.026	0	0	0.027	0
Lakes + rivers	0	0	0.087	0	0.089	0.134	0		0.003	0	0.035	0	0.016
Marine sediments	0.021	0.008	0.109	0.017	0.007	0.057	0.103	0.048		0	0.017	0.008	0.011
Seas	0	0	0.011	0.032	0	0	0.031	0	0.008		0	0.019	0
Soils	0	0	0.126	0	0.027	0.091	0	0.121	0.074	0		0	0.012
Ocean water column	0.025	0	0.050	0.071	0	0.007	0.113	0.007	0.038	0.075	0.003		0
Wastewater treatment	0.024	0	0.044	0	0.020	0.061	0	0.090	0.056	0	0.045	0	

*Values represent the Jaccard similarity index for (above the diagonal) *amoA* nucleotide OTUs at the 99% identity level and (below the diagonal) 95% identity level*.

**Table A2:** ***R* values for factors tested via ANOSIM for association with variation in archaeal *amoA* sequence diversity**.

Factor	Sequences included in analysis		
	Global alignment	Water column sequences	Water associated sequences (water column, groundwater, sea, lakes/rivers)
Habitat (13 levels)	**0.378** (*n* = 6202)	NA	NA
Latitude (low, mid, high)	**0.026** (*n* = 7138)	**0.296** (*n* = 440)	0.049 (*n* = 984)
Temperature (psychrophilic, mesophilic, thermophilic)	**0.170** (*n* = 2014)	**0.263** (*n* = 290)	NA
Water depth (surface, mid, deep)	NA	**0.464** (*n* = 898)	NA
Salinity (high, low)	NA	NA	**0.576** (*n* = 1402)
Marine versus freshwater / terrestrial^1^	**0.422** (*n* = 6202)	NA	NA
Soil/sediment versus aquatic	**0.099** (*n* = 6202)	NA	NA

*Significant *R* values (*P*<0.001) are indicated in bold. Level designations for each habitat are provided; definitions for levels are given in the text. The number of sequences included in each analysis (*n*) is given below *R* values. All factors are individually tested in ANOSIM analyses*.

*NA, not applicable*.

*^1^The marine level included sequences associated with aquaria and biofilters, coastal sediments, marine sediments, hydrothermal vents, seas, water column (marine), coral/sponge, and selected wastewater treatment sequences obtained from high-salinity bioreactors in Hong Kong (EU860262–EU860273, EU870438–EU870442). The terrestrial level included sequences associated with soils, lakes and rivers, caves, groundwater, hot springs, and the majority of the wastewater treatment sequences*.
